# Study of Multi-Objective Tracking Method to Extract Multi-Vehicle Motion Tracking State in Dynamic Weighing Region

**DOI:** 10.3390/s25103105

**Published:** 2025-05-14

**Authors:** Yan Zhao, Chengliang Ren, Shuanfeng Zhao, Jian Yao, Xiaoyu Li, Maoquan Wang

**Affiliations:** College of Mechanical Engineering, Xi’an University of Science and Technology, Xi’an 710054, China; 22205016035@stu.xust.edu.cn (C.R.); zsf@xust.edu.cn (S.Z.); 21205016032@stu.xust.edu.cn (J.Y.); 22205016036@stu.xust.edu.cn (X.L.); 22205224138@stu.xust.edu.cn (M.W.)

**Keywords:** weigh-in-motion, vehicle detection, trajectory extraction, accuracy compensation

## Abstract

Dynamic weighing systems, an advanced technology for traffic management, are designed to measure the weight of moving vehicles without obstructing traffic flow. These systems play a critical role in monitoring freight vehicle overloading, collecting weight-based tolls, and assessing the structural health of roads and bridges. However, due to the complex road traffic environment in real-world applications of dynamic weighing systems, some vehicles cannot be accurately weighed, even though precise parameter calibration was conducted prior to the system’s official use. The variation in driving behaviors among different drivers contributes to this issue. When different types and sizes of vehicles pass through the dynamic weighing area simultaneously, changes in the vehicles’ motion states are the main factors affecting weighing accuracy. This study proposes an improved SSD vehicle detection model to address the high sensitivity to vehicle occlusion and frequent vehicle ID changes in current multi-target tracking methods. The goal is to reduce detection omissions caused by vehicle occlusion. Additionally, to obtain more stable trajectory and speed data, a Gaussian Smoothing Interpolation (GSI) method is introduced into the DeepSORT algorithm. The fusion of dynamic weighing data is used to analyze the impact of changes in vehicle size and motion states on weighing accuracy, followed by compensation and experimental validation. A compensation strategy is implemented to address the impact of speed fluctuations on the weighing accuracy of vehicles approximately 12.5 m in length. This is completed to verify the feasibility of the compensation method proposed in this paper, which is based on vehicle information. A dataset containing vehicle length, width, height, and speed fluctuation information in the dynamic weighing area is constructed, followed by an analysis of the key factors influencing dynamic weighing accuracy. Finally, the improved dynamic weighing model for extracting vehicle motion state information is validated using a real dataset. The results demonstrate that the model can accurately detect vehicle targets in video footage and shows strong robustness under varying road illumination conditions.

## 1. Introduction

As road transportation demand increases sharply and the drive for higher economic efficiency intensifies, some transport operators have resorted to overloading their vehicles. While this practice may provide short-term economic benefits, it has long-term detrimental effects on road infrastructure, traffic safety, and the environment. Therefore, vehicle information detection is essential for effective road management.

Vehicle overload detection can be achieved through both static and dynamic weighing (WIM) methods. Static weighing technology is well-established and provides high detection accuracy [1], but its slower speed and complex on-site installation can lead to traffic congestion. In contrast, dynamic weighing allows for measurement of a vehicle while it is in motion, without the need for it to stop, significantly improving measurement efficiency [2]. However, the accuracy of dynamic weighing is relatively lower, which limits its widespread application. Consequently, enhancing the accuracy of dynamic weighing systems has become a crucial area of research [3,4].

To improve the accuracy of WIM systems, optimization is generally carried out in both hardware and software. In terms of hardware, the accuracy and stability of data collection can be enhanced by improving the load cell structure, processing methods, and its placement on the road surface. While many weighing algorithms focus on factors such as dynamic weighing signals and temperature, which affect accuracy, they often overlook the influence of the vehicle’s characteristics on weighing precision. In practical applications, coupled vibrations between the vehicle and the road during motion cause random fluctuations in the forces between them, leading to errors in the vehicle’s mass measured by dynamic load cells [5]. Additionally, the presence of multiple vehicles on the same road segment at a given time can alter the overall dynamic characteristics and behavior of each vehicle as it passes through the sensor [6], further impacting the accuracy of dynamic weighing. To enhance the accuracy of dynamic weighing systems, a comprehensive vehicle-road-weighing system model must be developed, with multi-target tracking playing a crucial role in accurately constructing the vehicle’s mathematical model [7,8].

Alex Bewley et al. [9] combined Kalman filtering with the Hungarian algorithm to propose the SORT tracking algorithm. Its core concept is to perform target tracking by jointly utilizing target detection and motion prediction. Known for its simplicity, efficiency, and real-time performance, the algorithm’s drawback is the frequent switching of vehicle IDs. A year later, the same team introduced the DeepSORT algorithm [10], which incorporates deep learning models to extract target appearance features and adds a cascade matching mechanism. This enhancement improves accuracy in situations with prolonged occlusion and significantly reduces the frequency of ID switching. The algorithm is now widely used in video surveillance, autonomous driving, and other applications. Li-Sheng Jin et al. [11] proposed an optimized vehicle multi-target tracking algorithm for DeepSORT, using Gaussian YOLO v3 as the front-end target detector. They introduced a new loss function that combines center loss with cross-entropy loss, which improves tracking accuracy and reduces identity switches compared to the baseline DeepSORT YOLO v3. Gao et al. [12] introduced an attention mechanism into the YOLO v5 network, combined with DeepSORT, and proposed the “double vertical line” algorithm. This method offers a new solution for repeated vehicle counting. Zhang et al. [13] proposed an improved monocular vehicle speed detection method that integrates YOLOX and DeepSORT. The method uses a YOLOX network enhanced with the ELAN module and CAENet attention mechanism for vehicle recognition, tracks vehicles using DeepSORT, and calculates the actual distances between frames through coordinate transformation to estimate vehicle speed.

Multi-target tracking refers to the system’s capability to simultaneously detect and track multiple vehicles or their components (e.g., axles) as they pass through the weighing equipment, ensuring accurate alignment of each vehicle’s speed, axle spacing, and weight information. This process typically involves the following aspects:

Tracking multiple vehicles: When several vehicles enter the weighing area nearly simultaneously, the system must distinguish and continuously track each one to avoid data overlap or confusion. Tracking axles or tires: To achieve precise vehicle and axle weight measurements, the movement trajectories of each vehicle’s axles must be accurately tracked. Matching weighing data with image/video streams: This involves establishing a one-to-one correspondence between the trajectory data captured by cameras and the weight data recorded by the weighbridge [13].

Multi-target tracking techniques are primarily categorized into online real-time tracking and offline tracking [13]. In the dynamic weighing scenario discussed in this paper, the traffic camera captures about 3 s of video as the vehicle passes through the dynamic weighing area. Although the video information is analyzed after collection, the vehicle’s weighing results must be displayed in real-time on the LED screen by the roadside for dynamic weighing accuracy compensation. To achieve this, all processing must be completed within a short time frame, which is why this paper uses an online multi-target tracking algorithm.

A review of existing literature reveals that current research lacks a method capable of simultaneously measuring vehicle dimensions and accurately identifying frequently switching vehicle IDs. To address this gap, this paper proposes an improved SSD vehicle detection model that mitigates the high sensitivity to vehicle occlusion and the frequent ID changes observed in existing multi-target tracking methods. First, the proposed model aims to reduce detection omissions caused by vehicle occlusion. Second, to obtain more stable trajectory and speed data, the Gaussian Smoothing Interpolation (GSI) method is integrated into the DeepSORT algorithm. Finally, the performance of the improved model in vehicle tracking tasks within dynamic weighing areas is validated through a series of experiments.

This paper analyzes the dataset collected from a dynamic weighing monitoring station, investigating the correlation between traffic flow speed, vehicle dimensions (length, width, and height), and weight distribution. The average speed, coefficient of variation in speed, and root mean square of acceleration of vehicles passing through the dynamic weighing area were used to assess speed fluctuations. PCA-based data analysis methods were then employed to analyze the dynamic weighing dataset, vehicle dimensions, and motion state information [14]. The results indicate that vehicle weight is strongly correlated with fluctuations in vehicle speed and is also moderately correlated with vehicle length. Finally, statistical and experimental methods were used to analyze the collected data, and accuracy compensation for dynamic weighing results was applied based on the root mean square of acceleration for vehicles passing through the dynamic weighing area. This approach effectively improved the dynamic weighing system’s accuracy to within 1.8%, which will facilitate the development of personalized corrections for the system.

## 2. Methods

### 2.1. Vehicle Multi-Target Tracking Algorithm Idea

**Figure 1:**
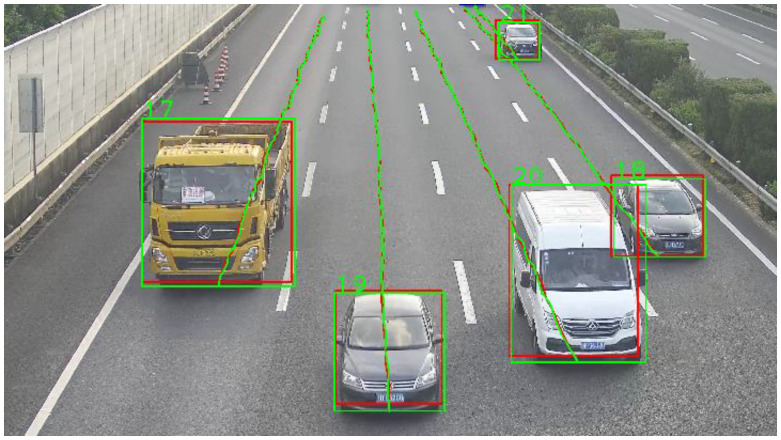
Vehicle video multi-target tracking. (Green for model features, red for motion features).

**Figure 2:**
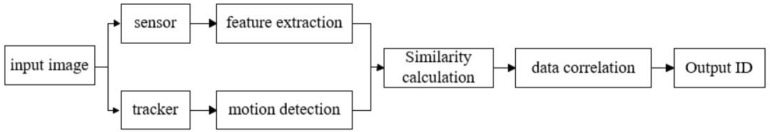
Flow chart of multi-target tracking.

The strategy for extracting vehicle motion state information within the dynamic weighing zone involves a multi-objective tracking method using DeepSORT and SSD [15]. Specifically, the SSD model is chosen for target detection, and its loss function is optimized to reduce detection accuracy degradation caused by vehicle occlusion [16]. To accurately track multiple targets, the DeepSORT algorithm is employed, which predicts target trajectories using Kalman filtering [17] and correlates detected targets with trajectories using the Hungarian algorithm and cascade matching strategy [18]. To enhance the smoothness and stability of the output trajectories, Gaussian Smoothing Interpolation (GSI) is applied to the trajectory data for post-processing. Finally, the effectiveness of the proposed method in extracting the motion state information of vehicles passing through the dynamic weighing zone is validated through experiments (Figure 1 and Figure 2).

### 2.2. Motion State Extraction Information for Multiple Vehicles

To accurately extract the motion state of vehicles passing through the dynamic weighing area, the primary task is to analyze approximately 3 s of surveillance video for vehicle detection and trajectory tracking. First, vehicles are identified in each video frame using the SSD model, a popular network for object detection. The detected vehicles are then tracked by assigning unique IDs to each target, recognizing them in subsequent frames to generate tracking trajectories. Multi-target tracking typically uses two strategies to determine if targets in different frames are the same: one is based on the target’s motion characteristics, predicting its position in the next frame based on its previous trajectory; the other is based on the target’s appearance characteristics. Current methods usually combine both motion-based and appearance-based strategies, rather than relying on one alone. This combined approach, known as detection-based tracking, performs target detection in each frame and uses data association techniques to match the detected targets. This paper uses the DeepSORT online multi-target tracking algorithm, which is known for its high accuracy and robustness, and is widely used in vehicle tracking. After vehicle detection and tracking are completed, obtaining trajectory and speed information requires camera calibration. This allows coordinate transformation and establishes a precise point-to-point mapping from vehicle coordinates in the image coordinate system to road surface coordinates in the world coordinate system. This paper uses a GPS-based camera calibration method.

### 2.3. Model Building

To accurately extract the vehicle’s motion state in the dynamic weighing area, it is essential to detect the vehicle in each video frame. Analysis of the surveillance video reveals that vehicle tracking in the weighing area primarily involves medium and large targets that move toward the camera, increasing in size. There are no small or hard-to-detect vehicle targets. However, achieving high detection accuracy is crucial for effective tracking, as insufficient accuracy may cause noticeable jitter when extracting vehicle trajectories within the weighing area.

SSD is a leading deep learning-based object detection framework [19], which combines the anchor-based mechanism and feature pyramid structure (FPN) from Faster R-CNN with the direct regression method of YOLO. This allows it to predict an object’s bounding box and category directly through an end-to-end network [20]. Additionally, SSD eliminates the fully connected layers, significantly improving detection accuracy compared to YOLO networks. However, SSD’s performance still needs improvement when handling partially occluded objects. In dynamic weighing processes, especially when vehicle density is high, mutual occlusion between vehicles is inevitable, further affecting detection accuracy. Therefore, this paper proposes improvements to SSD to enhance both its detection accuracy and speed.

**Figure 3:**
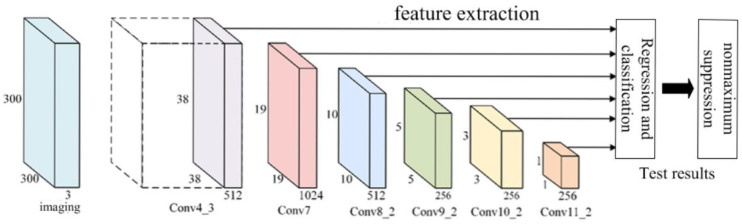
SSD Network Diagram.

The SSD network model architecture consists of three main components: the base network, feature extraction network, and detection network. This model improves the classical VGG16 to suit target detection tasks. To preserve the full spatial feature location information and avoid the spatial abstraction of fully connected layers, SSD replaces the last two fully connected layers of VGG16 with convolutional layers, named Conv6 and Conv7. The model then introduces four additional convolutional layers—Conv8, Conv9, Conv10, and Conv11—alternating between different kernel sizes (1 × 1 and 3 × 3) to downscale and extract features. A multi-scale feature network is created by fusing feature maps from Conv4_3 and Conv7, along with subsequent layers (Conv8_2, Conv9_2, Conv10_2, and Conv11_2), forming a feature pyramid structure [21]. In the detection network, each layer’s feature maps are processed with two 3×3 convolutional kernels: one for predicting target category confidence and another for generating positional regression information. These outputs are aggregated and fed into the loss layer for optimization, with the final detection results refined through non-maximum suppression (NMS). Figure 3 illustrates the basic structure of the SSD network model.

The SSD model predicts an object’s position at different scales and aspect ratios using a series of fixed-size anchor boxes. These anchor boxes serve as candidate regions across multiple feature maps, covering various positions and sizes within the input image. The SSD regression mechanism consists of two main steps: anchor box matching and bounding box regression. During the anchor box matching phase, the model calculates the intersection-over-union (IoU) to find the most compatible ground truth box (GTB) for each anchor box [22]. This matching process ensures that each anchor box is linked to the closest ground truth box. Once matching is complete, the model enters the bounding box regression phase, where the objective is to learn the offset between the anchor box and the true bounding box. These offsets include adjustments to the object’s center position and changes in the bounding box dimensions. Through this process, the SSD model can more accurately predict the exact bounding box of each target object. The SSD loss function consists of confidence loss and localization loss. The localization loss evaluates the deviation between the predicted and actual bounding box locations, while the confidence loss measures the model’s confidence in the predicted category of the bounding box. The total loss function of SSD is the weighted sum of the localization and confidence losses, defined as:

(1) $$L(x,c,l,g)=\frac{1}{N}(L_{conf}(x,c)+\alpha L_{loc}(x,l,g))$$

where *N* represents the number of pre-checked boxes that match the same true box.

In this paper, we introduce exclusion loss in addition to the original position and confidence losses. Exclusion loss is calculated based on the overlap rate between the detection box and surrounding non-vehicle objects. Its purpose is to minimize the overlap with non-vehicle objects, thereby improving the detection box’s accuracy in localizing vehicle objects. The improved loss function is expressed by the following equation:

(2) $$L=L(x,c,l,g)+\gamma L_{Rep}GT$$

$L=L(x,c,l,g)+\gamma L_{Rep}GT$ where $L_{Rep}GT$ is the excluded loss and γ is the weighting factor used to balance the ancillary losses.

Use set $P_{+}=\left\{P\right\}$ to denote all candidate frames *P* whose intersection and concurrency ratios with real frames have IoU greater than 0.5. Set $G_{+}=\left\{G\right\}$ contains all the real frames in the image. For a candidate frame *P*, find the real frame with the largest IoU among all real frames *G*, denoted as $G_{Attr}^{P}$, i.e.:

(3) $$G_{Arrt}^{P}=\underset{G\in G_{+}}{arg\max}IoU(G,P)$$

In exclusion loss, an exclusion object is defined, and the exclusion object is the one real frame that has the next largest IoU value with P among all real frames (except the specified object), which can be described by the following mathematical expression:

(4) $$G_{Rep}^{P}=\underset{G\in G_{+}\left\{G_{Attr}^{P}\right\}}{arg\max}IoU(G,P)$$

$G_{Rep}^{P}$ stands for excluded objects. i.e., the real frame with the largest IoU between it and the candidate frame P except for the specified object $G_{Atrr}^{P}$. $G_{+}$ is the set of all real frame, while $G_{+}\left\{G_{PAttr}\right\}$ represents the set after removing the specified object from all real frame. IoU(G,P) calculates the intersection and concurrency ratio between candidate frame P and real frame G. $B^{P}$ is the final detection frame predicted by the model based on the candidate frame *P* and the learned offset, which are intended to closely cover the real objects in the image. The overlapping IoG between $B^{P}$ and $G_{Rep}^{P}$ can be expressed by the following equation:

(5) $$IoG\left(B^{P},G_{Rep}^{P}\right)=\frac{area\left(B^{P}\cap G_{Rep}^{P}\right)}{area\left(G_{Rep}^{P}\right)}$$

where $area(B^{P}\cap G_{PRep}^{P})$ represents the area of the overlapping region of the detection frame $B_{P}$ and the exclusion object, $area(G_{PRep})$, is the area of the exclusion object $G_{PRep}$.

In summary, the excluded loss can be calculated using the following formula:

(6) $$L_{RepGT}=\frac{IoG(B^{P},G_{Rep}^{P})}{\left|P_{+}\right|}$$

### 2.4. DeepSORT Algorithm

**Figure 4:**
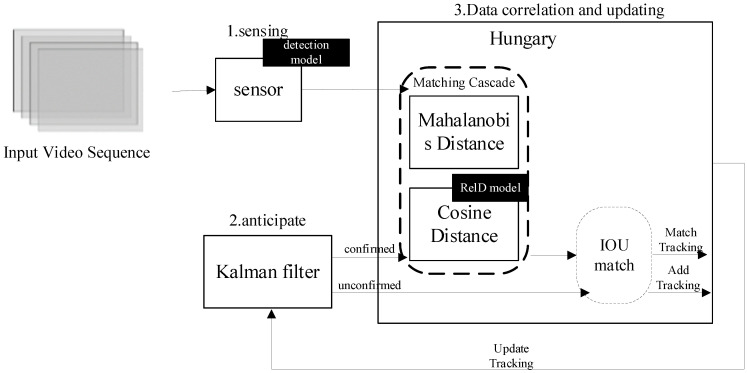
Flowchart of DeepSORT algorithm.

To extract the motion state of vehicles passing through a dynamic weighing area, vehicle tracking is required. With advances in target detection technology, detection-based tracking methods have become the mainstream in multi-target tracking. The SORT algorithm combines the Kalman filter with the Hungarian algorithm, using the intersection-over-union (IoU) as the cost matrix in the Hungarian algorithm, offering an efficient tracking strategy. However, this algorithm overlooks the role of visual features in target matching, which can lead to identity switch errors during occlusion and disrupt tracking continuity [23]. To address this issue, the DeepSORT algorithm integrates dynamic and appearance information as matching criteria and employs a cascade matching strategy, effectively reducing identity switches and tracking loss [24], as shown in Figure 4.

The first step of the DeepSORT algorithm is target detection, where each frame is processed by the improved SSD algorithm to identify vehicles. Once a vehicle is detected, the algorithm enters the prediction phase, and uses a Kalman filter to predict the vehicle’s position in the next frame based on previous motion data. The initial detection results set the state variables for the Kalman filter. Predicted trajectories are classified as “confirmed” or “unconfirmed” based on their match with the detection results. Newly generated trajectories start as unconfirmed and only become confirmed after matching with the detection frame a set number of times. If a confirmed trajectory fails to match a detection frame for several consecutive frames and exceeds the maximum allowed unmatched frames (max age), the trajectory is removed from the system. After prediction, the detected and predicted vehicles are matched. DeepSORT uses a Hungarian algorithm that combines Mahalanobis distance and cosine distance of appearance features in a cascade matching strategy to find the optimal match. Mahalanobis distance measures the distance between motion states, and if it is below a predetermined threshold, the match is considered successful. The cosine distance of appearance features is computed by a re-identification model to assess target similarity. When the combined cost function of both distances is small—i.e., when the predicted and detected frames are close in both spatial location and appearance features—they are considered the same target. This cascade method addresses potential inaccuracies when relying solely on motion states for matching, especially during drastic motion changes. When motion changes are minimal, Mahalanobis distance serves as an effective data association metric. For unmatched prediction frames, further association attempts are made by calculating their intersection-over-union (IoU) with the detection frames. If the IoU is below a specific threshold, the frames are not associated. Finally, based on the current frame’s association results, the states of all tracked objects with unique identifiers (IDs) are updated to maintain tracking continuity and accuracy. Through these steps, the DeepSORT algorithm efficiently tracks targets in the video, maintaining high tracking accuracy even in fast-moving targets or complex scenes.

When target loss occurs (e.g., due to occlusion or detection failure), DeepSORT uses linear interpolation to predict the target’s next position. This simple interpolation method does not account for the target’s motion pattern, which can lead to inaccurate trajectory predictions, especially when the target’s speed or trajectory curvature changes significantly [25]. To improve the accuracy of vehicle speed recognition, this paper optimizes DeepSORT by using the Gaussian smoothing interpolation (GSI) algorithm. GSI uses Gaussian process regression (GPR) to predict the target’s position, considering both motion information and historical trajectories. By performing nonlinear interpolation based on the target’s motion pattern, GSI provides smoother and more accurate trajectory estimates compared to simple linear interpolation, thereby improving the accuracy of velocity and acceleration estimation.

The GSI model for the *i* trajectory can be expressed as follows:

(7) $$p_{t}=f^{\left(i\right)}\left(t\right)+\varepsilon$$

where t∈F is the number of frames, $p_{t}\in P$ is the motion coordinates at frame *t*, and $\varepsilon ∼N\left(0,\sigma^{2}\right)$ is the Gaussian noise.

For a given trace of length L, the linearly interpolated trajectory is:

(8) $$S^{\left(i\right)}={\left\{t^{\left(i\right)},p_{t}^{\left(i\right)}\right\}}_{t=1}^{L}$$

The problem of modeling nonlinear motion is solved by fitting a function $f\left(i\right)$ that is assumed to obey a Gaussian process:

(9) $$f^{\left(i\right)}\in GP\left(0,k\left(\cdot ,\cdot \right)\right)$$

where $k\left(x,x\prime \right)=\exp\left(-\frac{{\left\|x-x\prime \right\|}^{2}}{2\lambda^{2}}\right)$ is the radial basis function kernel.

According to the nature of the Gaussian process, given a new set of frames $F^{*}$, its smoothing position $P^{*}$ is predicted by the following equation:

(10) $$P^{*}=K\left(F^{*},F\right){\left(K\left(F,F\right)+\sigma^{2}I\right)}^{-1}P$$

where $P^{*}$ is the predicted smoothed position on the new set of time points $F^{*}$ and *P* is the set of known positions on the original trajectory.$K(F^{*},F)$ is a covariance matrix representing the interactions between the new set of time points $F^{*}$ and the original set of time points *F*, K(F,F) is the covariance matrix for the interactions between the original time points, and $\sigma^{2}I$ is the covariance term of the observation noise.

The hyperparameter λ, which controls the degree of smoothness of the trajectory, is designed as an adaptive function dependent on the length *l* of the trajectory, in the specific functional form:

(11) $$\lambda =\tau \times \log\left(\frac{\tau^{3}}{l}\right)\;$$

where τ is a preset constant to adjust the basis size of λ, which is set to 10 in this paper. This hyperparameter directly affects the nature of the kernel function k(⋅,⋅), and thus adjusts the degree of trajectory smoothing. When the trajectory is short, the value of λ is larger, which leads to higher smoothness and helps to reduce trajectory fluctuations due to noise. Conversely, for longer trajectories, the value of λ decreases and the smoothness decreases, which allows the trajectory to better reflect subtle changes in the target’s motion.

### 2.5. Algorithmic Fusion Coordinate Transformation

**Figure 5:**
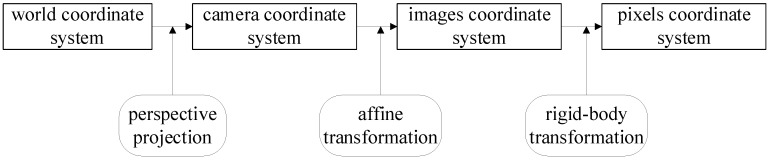
Camera linear model conversion process.

The vehicle tracking method based on SSD and DeepSORT proposed in this paper extracts the trajectory and speed information of the vehicle. To achieve this, the vehicle’s position in the pixel coordinate system must be converted to real-world coordinates. We employ a GPS-based camera calibration method to map the target’s position on the camera screen to its real-world position. Specifically, the distance the vehicle moves per unit time on the screen is mapped to the actual distance, allowing the calculation of speed and trajectory information [26]. As shown in Figure 5, the method involves transformations between the world coordinate system, camera coordinate system, image coordinate system, and pixel coordinate system.

This paper employs a GPS-based method for calibrating camera parameters. By using GPS-provided coordinates instead of traditional calibration templates, this method effectively reduces errors caused by template inaccuracies. The calibration process involves the following steps: First, four distinct locations on the road are selected, and the GPS coordinates and corresponding image data for these locations are recorded. Next, the coordinates are converted into the world coordinate system based on the relationship between the GPS and world coordinate systems. Finally, the camera parameter matrix is computed using the camera imaging model and the collected data.

To establish the world coordinate system, a reference point is chosen as the origin (O), with the x-axis directed north, the y-axis directed east, and the z-axis perpendicular to the ground. This forms a local coordinate system tangent to the Earth’s surface. Since the road surface in front and behind the dynamic weighing system is typically flat, this paper defines the section of the road as the plane z = 0 and converts the GPS coordinates of all points to this coordinate system for subsequent transformation and analysis.

First, the GPS coordinate system is converted to the Earth’s Cartesian coordinate system with the following formula:

(12) $$X_{E}=N\cos B\cos L$$

(13) $$Y_{E}=N\cos B\sin L$$

where *B*, *L* represent the longitude and dimension of any position in the coordinate system, respectively, $X_{E}$, $Y_{E}$ are the coordinates of the corresponding points in the Cartesian coordinate system of the Earth, *N* is the radius of curvature of the ellipsoid, and *E* is the first eccentricity of the ellipsoid. The long radius of the Earth, a, 6,378,137 m and the short radius, b, is 635,675 m.

(14) $$E=\frac{\sqrt{a^{2}-b^{2}}}{a}$$

(15) $$N=\frac{a}{\sqrt{1-E^{2}\sin^{2}B}}(B,L)$$

Next, in order to convert points in the Earth’s Cartesian Coordinate System to the World Coordinate System, the following conversion formula is applied:

(16) $$X=X_{E0}-X_{E}$$

(17) $$Y=Y_{E0}-Y_{E}$$

The camera imaging model is shown below:

(18) $$s_{i}\left[\begin{array}{c} u_{i}\\ v_{i}\\ 1 \end{array}\right]=\left[\begin{array}{ccc} m_{11} & m_{12} & m_{13}\\ m_{21} & m_{22} & m_{23}\\ m_{31} & m_{32} & m_{33} \end{array}\right]\left[\begin{array}{c} X_{i}\\ Y_{i}\\ 1 \end{array}\right]$$

where s_i_ is the scale factor, α is the coordinates of the different positions in the world coordinate system, β is the coordinates of the corresponding points in the image coordinate system, and *M* is the camera parameter matrix to be solved. By using the known world coordinates of the four positions and their corresponding coordinates in the image coordinate system, the camera parameter matrix *M* can be solved using a direct linear transformation method.

(19) $$\alpha =\left[\begin{array}{c} X_{i}\\ Y_{i}\\ 1 \end{array}\right]$$

(20) $$\beta =\left[\begin{array}{c} u_{i}\\ v_{i}\\ 1 \end{array}\right]$$

(21) $$M=\left[\begin{array}{ccc} m_{11} & m_{12} & m_{13}\\ m_{21} & m_{22} & m_{23}\\ m_{31} & m_{32} & m_{33} \end{array}\right]$$

**Figure 6:**
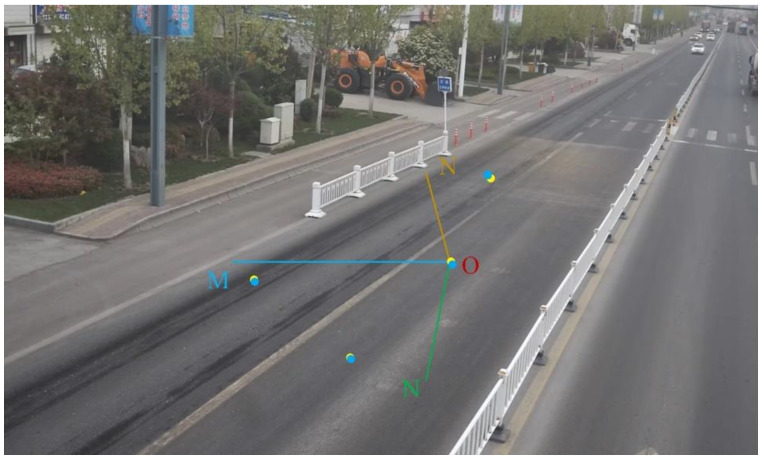
Pavement coordinate system and reference points.

As shown in Figure 6, the blue points (pixel coordinates) and the yellow points (pixel coordinates of the pavement locations) align closely on the image after projection transformation. This indicates that the projection transformation method effectively maps actual pavement coordinates to the image coordinate system. To verify the accuracy of the transformation, we compared the distances between the four measured points with those obtained through the transformation. The results show that the maximum coordinate error is 0.194 m, with a relative error of 1.27%. This error range is acceptable for vehicle motion state estimation and meets the required accuracy.

## 3. Production of Data Sets

### 3.1. Sources of Vehicle Datasets

The vehicle label and axle type detection model requires a comprehensive dataset containing various detection targets to achieve high recognition accuracy. The detection algorithms for vehicle labels and axle types are based on actual vehicle dimensions, requiring images from different perspectives and types. Images of vehicle labels and axle types, captured from various scenes and of different sizes, are adapted and improved to enhance the model’s robustness. In this study, most of the image data for vehicle labels and axle types in the detection dataset are sourced from surveillance videos of the integrated highway control system in Xiaolei Village, Yaozhou District, Tongchuan City, Shaanxi Province. Images of vehicle labels for some vehicle types are collected from the internet, ensuring sufficient training data for the model.

**Figure 7:**
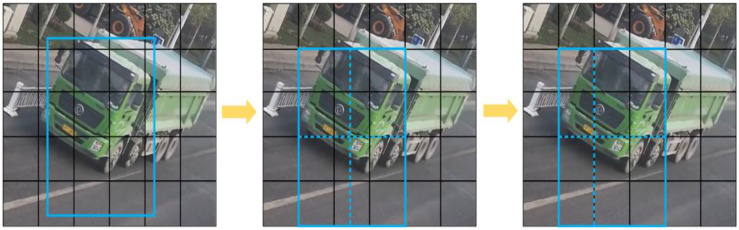
LableImg Labeling of Vehicle Markings and Axle Types.

The surveillance video was converted into single-frame images using Matlab2022a, after which unclear images were removed, resulting in a total of 5621 relevant images. The dataset was then created in VOC format using the LableImg tool [27]. The dataset was split into training and test sets in a 9:1 ratio. The LableImg interface for annotating the vehicle label and axle type detection dataset is shown in Figure 7.

Through on-site research and analysis of the traffic flow in the road section’s surveillance video, four axle types and seven vehicle label categories were identified. Due to the scarcity of five-axle vehicles and the complexity of small car labels, the dataset excludes five-axle vehicles, and all seven label types correspond to truck labels. The distribution of different vehicle labels and axle types in the dataset is shown in Table 1.

**Figure 8:**
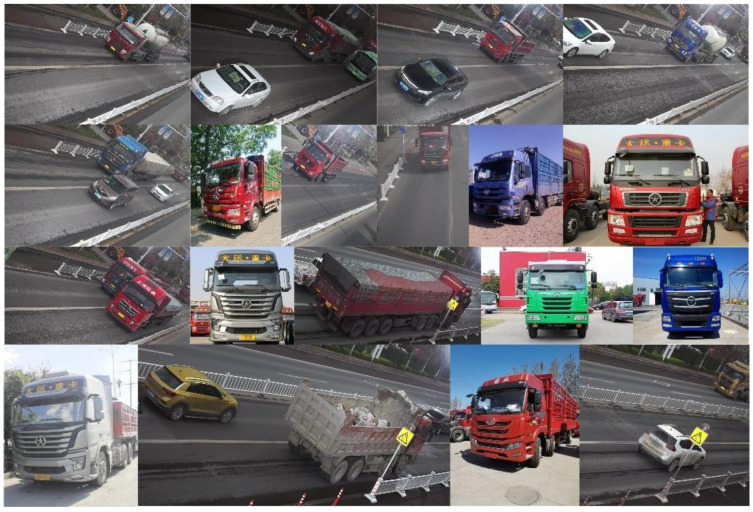
Sample of some images in the marking and axle type detection dataset.

As shown in Table 1, the dataset contains multiple types, but the amount of data for each type is insufficient. Additionally, the data for the two-axis axle type is uneven compared to other types, resulting in a data imbalance. During the training of the vehicle label and axle type detection network, this imbalance can cause the model to be biased toward the types with larger datasets, while the types with fewer data may lead to poor generalization of the final model, affecting its detection accuracy. To address this issue, the vehicle label and axle type datasets were expanded using image data augmentation. Figure 8 shows some image samples from the dataset.

### 3.2. Training of Detection Models

The vehicle size measurement network was trained using a custom dataset containing 18,400 images, with 12,000 for training, 3680 for testing, and 2720 for validation. During the network training phase, the SGD optimizer was used to update the network’s weights, with an initial learning rate of 0.0001, a momentum of 0.9, a weight decay of 0.001, a batch size of 8, and 100 epochs. The training environment is described in Table 1 above.

To evaluate the network model’s performance in predicting vehicle dimensions, this study uses several metrics, including Mean Absolute Error (MAE), Root Mean Square Error (RMSE), FLOPs, and the number of model parameters. MAE measures prediction accuracy by calculating the absolute difference between each predicted and true value for length, width, and height, and is insensitive to outliers. RMSE calculates the square of the differences between predicted and true values for the three dimensions, averages the squared differences, and takes the square root, giving higher weight to larger prediction errors. FLOPs reflect the computational load required by the model during training, while the number of parameters is directly related to the model’s storage needs and computational burden. Together, these two metrics provide detailed insights into the model’s computational resource consumption. The formulas for MAE and RMSE are as follows:

(22) $$MAE=\frac{1}{3N}\sum_{i=1}^{N}\left(\left|L_{i}-l_{i}\right|+\left|H_{i}-h_{i}\right|+\left|W_{i}-w_{i}\right|\right)$$

(23) $$RMSE=\sqrt{\frac{1}{3N}\sum_{i=1}^{N}\left[{\left(L_{i}-l_{i}\right)}^{2}+{\left(H_{i}-h_{i}\right)}^{2}+{\left(W_{i}-w_{i}\right)}^{2}\right]}$$

where $L_{i},W_{i},H_{i}$ denotes the true aspect and $l_{i},w_{i},h_{i}$ denotes the predicted aspect.

**Figure 9:**
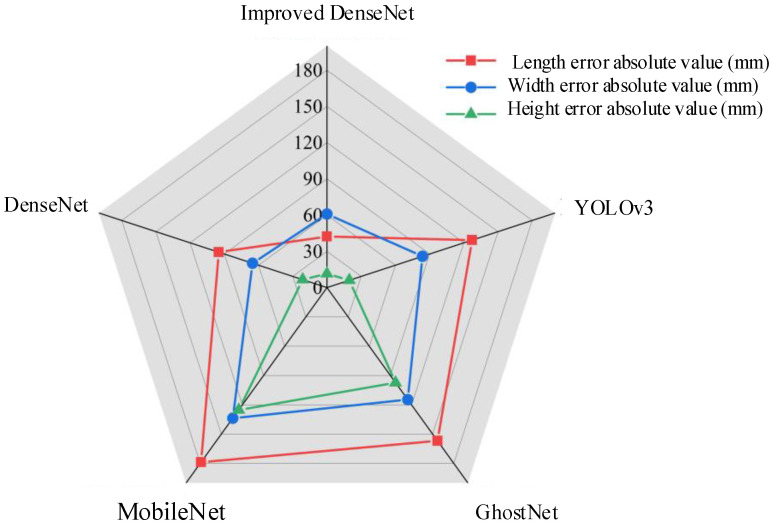
Errors in predicting Tesla by different models.

To verify the performance advantages of the improved DenseNet, a comparison is made with other commonly used network models on the same dataset. Table 2 presents the loss function, FLOPs, and the number of parameters for YOLOv3, MobileNetV1, GhostNet, DenseNet, and the improved DenseNet during training. The model proposed in this paper achieves the highest recognition accuracy. Although its number of parameters and FLOPs are not as efficient as the lightweight MobileNet and GhostNet, it shows significant advantages over YOLOv3 and the original DenseNet. Furthermore, it reduces the network’s parameter count and increases speed while maintaining high accuracy. Figure 9 shows the absolute errors of the Tesla Model 3 predictions by the five models on the same dataset, with the proposed model outperforming the others in terms of accuracy.

**Figure 10:**
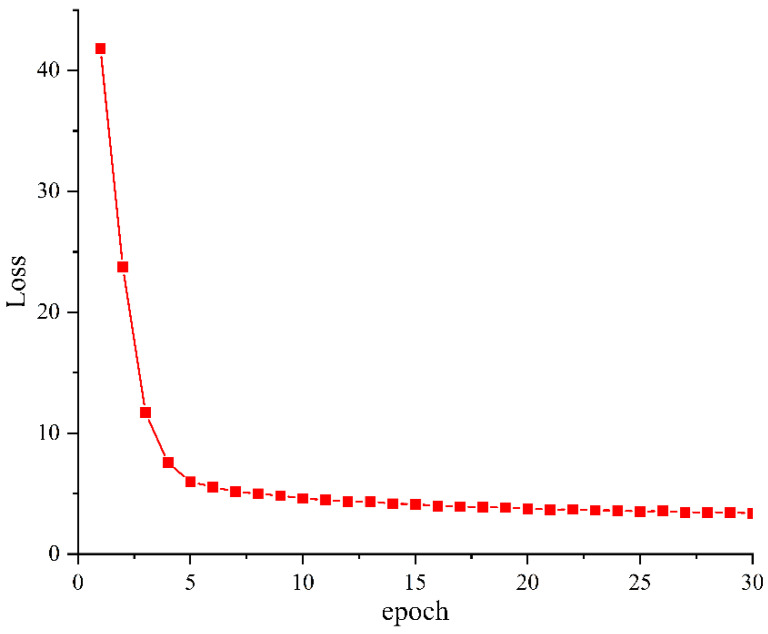
Variation curve of training loss of vehicle marking and axle type detection models.

During the training of the vehicle label and axle type detection model, the changes in the model’s training loss function indicate whether the model has reached convergence. Figure 10 shows the loss curve during training. As seen from the graph, the training loss rapidly decreases in the first five epochs. From the fifth to the fifteenth epoch, the reduction in loss slows down, and from the fifteenth to the thirtieth epoch, the loss stabilizes and the model reaches near completion.

### 3.3. Data Set Sampling Results and Analysis

To verify the performance of the detection algorithm proposed in this paper, it is compared with traditional detection algorithms such as R-CNN, Fair MOT, and YOLOv3 [28] using the test set of the vehicle label and axle type detection models, based on the trained detection models.

To evaluate the detection and recognition performance of vehicle mark and axle type detection, Accuracy and Recall are used as metrics. Recall is the proportion of correctly detected samples among all relevant samples, while Accuracy is the proportion of correctly detected samples among all detected samples. They are defined by the following equations respectively:

(24) $$Accuracy=\frac{TP+TN}{TP+TN+FP+FN}$$

(25) Recall=TPTP+FN

In this context, TP denotes the number of targets in the image that are true targets and are correctly detected by the model; TN denotes the number of targets in the image that are true targets but are incorrectly detected by the model; FP denotes the number of targets in the image that are not true targets but are incorrectly detected as belonging to the detection category by the model; and FN denotes the number of targets in the image that are not true targets and are incorrectly detected as not belonging to the detection category by the model.

As shown in Table 3, the vehicle mark and axle type detection model proposed in this paper achieves higher detection accuracy and recall than other algorithms, demonstrating its superior detection performance and speed.

**Figure 11:**
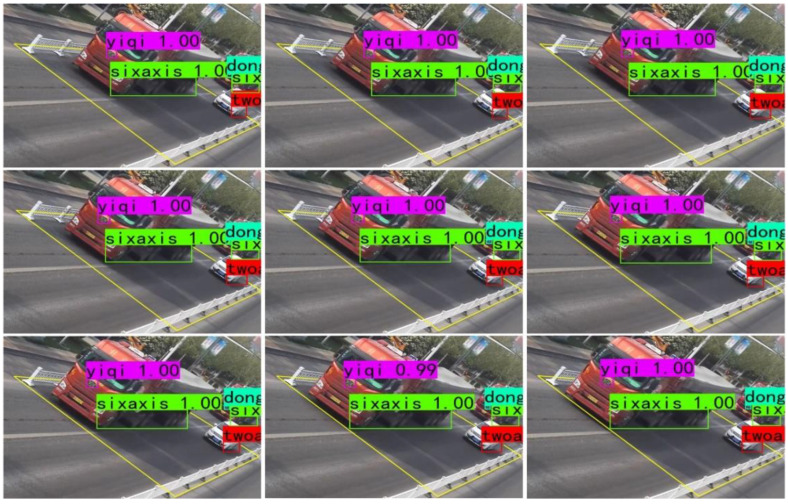
The effect of detecting vehicles in the dynamic weighing area in nine consecutive frames.

To verify that the detection model performs well in other time periods of the dynamic weighing area, surveillance video data from different periods were re-collected for experimental validation. The results show that the model accurately detects the vehicle label and axle type of vehicles passing through the dynamic weighing area, with no omissions or misdetections. As shown in Figure 11, the detection results for nine consecutive frames of a vehicle passing through the dynamic weighing area are presented.

## 4. Experimental Results and Analysis

### 4.1. Data Set Correlation Analysis

After analyzing the dataset from the dynamic weighing system, it was found that the weighing results may be influenced by the vehicle’s speed and length when passing through the dynamic weighing area [29]. To verify this relationship, this paper analyzes the correlation between vehicle dimensions (length, width, and height), the vehicle’s speed and speed fluctuation while passing through the dynamic weighing area, and dynamic weighing accuracy. Since the DeepSORT algorithm detects the vehicle’s speed at different moments as it passes through the dynamic weighing area [30], this complicates the data analysis. To simplify and enhance the analysis, the vehicle’s average speed, the coefficient of variation of speed, and the root mean square of acceleration are used to describe the vehicle’s speed variation.

The average speed directly reflects the overall speed level of the vehicle as it passes through the dynamic weighing zone. The Coefficient of Variation (CV), calculated as the standard deviation divided by the mean and multiplied by 100%, helps assess the degree of fluctuation and is scale-invariant. The CV for speed is the ratio of the speed’s standard deviation to its mean, reflecting the relative fluctuation of speed around its average value. If the CV of the vehicle’s speed through the dynamic weighing zone is high, it indicates that the vehicle’s speed is highly variable, i.e., there is significant fluctuation. Since a vehicle may accelerate and then decelerate, or decelerate and then accelerate as it passes through the dynamic weighing zone, resulting in positive and negative changes in acceleration, the CV is used to measure the dispersion of positive values. However, for acceleration, which can have both positive and negative values, using the CV does not fully capture its significance. Therefore, this paper uses the Root Mean Square (RMS) to describe the fluctuation of acceleration. A higher RMS value indicates greater variation or fluctuation in acceleration. The formulas for both are as follows:

(26) $$CV=(\frac{\sigma_{v}}{\mu_{v}})\times 100\%$$

(27) $$RMS=\sqrt{\frac{1}{n}\sum_{i=1}^{n}a_{i}^{2}}$$

where $\sigma_{v}$ is the standard deviation of the velocity, $\mu_{v}$ is the mean value of the velocity, and $a_{i}$ is the acceleration calculated from the root velocity.

In this paper, six variables are selected for comprehensive analysis: vehicle length, width, height, average speed, speed variation coefficient, and acceleration variation coefficient. The analysis investigates whether these variables affect the dynamic weighing results. The PCA (Principal Component Analysis) algorithm is a commonly used statistical method that transforms the original data into a new coordinate system through linear transformation, maximizing the variance of the data in this new system. During this process, the eigenvalues and eigenvectors of the covariance matrix are computed to determine the direction of the principal components, and the first K principal components are selected based on their variance contribution.

The algorithmic flow of PCA is:

The input is the n-dimensional sample set $C=(x^{\left(1\right)},x^{\left(2\right)},\ldots ,x^{\left(m\right)})$, reduced to $n^{\prime }$ dimensions; the output is the reduced sample set $C^{\prime }$.

(1) Centering the sample, even if the mean of each feature is 0:

(28) $$x^{\left(i\right)}=x^{\left(i\right)}-1/m\sum_{j=1}^{m}x^{\left(j\right)}$$

(2) Calculate the covariance matrix of the sample set and decompose it into eigenvalues in order to find the eigenvalues of the covariance matrix and the corresponding eigenvectors;
(3) The eigenvectors $\left(w_{1},w_{2},\ldots ,w_{n}\right)$ corresponding to the largest $n^{\prime }$ eigenvalue are taken out and these eigenvectors are normalized to form the eigenvector matrix W;
(4) For each sample $x^{\left(i\right)}$ in the sample set, transform into a new sample $z^{i}=W^{T}x^{\left(i\right)}$;
(5) Get the output sample set $C^{\prime }=(z^{\left(1\right)},z^{\left(2\right)},\ldots ,z^{\left(m\right)})$.

The PCA algorithm was used to analyze the six extracted features that may affect the dynamic weighing results, the analyzed dimensions were selected according to the contribution of each principal component, and the corresponding contribution rates and cumulative contribution rates are shown in Table 4 below.

In PCA analysis, the contribution rate measures the importance of each principal component in preserving the original data information [31]. Generally, if the contribution rate of a component exceeds a predetermined threshold, it is considered significant and should be included; conversely, components with lower contribution rates can be discarded. When performing PCA analysis, a clear target threshold for the cumulative contribution rate is typically set, usually at $M_{m}\geq 85\%$. As shown in Table 4, the cumulative contribution rate of the first four principal components reaches 97.1%, indicating that these components effectively capture the core features of the data and retain most of the key information. Therefore, it can be concluded that the main factors affecting the dynamic weighing results are the vehicle’s speed as it passes through the dynamic weighing area, speed fluctuations, and the vehicle’s length, although the length has a smaller impact.

### 4.2. Analysis of Dynamic Weighing System Data Sets

The dynamic weighing system dataset includes traffic flow data from a monitoring station over one working day. Based on the vehicles with different axle counts identified by the system, the average length, width, height, and speed distributions of these vehicles were statistically analyzed. Table 5 presents the average dimensions, speed, number of vehicles, and average weight distribution of vehicles with different axle counts.

It can be seen that two-axle vehicles have smaller average length, width, height, and mass. This is because two-axle vehicles are mainly domestic cars, with two-axle trucks representing a smaller proportion compared to domestic cars, leading to higher average speeds for two-axle vehicles passing through the dynamic weighing area. Three-axle, four-axle, and six-axle vehicles dominate freight transportation. Three-axle vehicles primarily transport construction materials and minerals, mostly in the form of tippers, but also include some special-purpose vehicles, such as flatbed and tank trucks, to accommodate more diverse transportation needs. Four-axle vehicles are mainly used for heavier-duty tippers and tractor-trailers to meet higher load requirements. six-axle vehicles are typically combinations of tractor-trailers and trailers, designed for major transportation tasks. Due to the variety of trailer types and their purposes, it is difficult to determine the full length of six-axle vehicles directly from manufacturers.

According to GB1589-2016 [32], “Outline Dimensions, Axle Load and Mass Limits for Vehicles, Trailers, and Vehicle Trains,” various types of trucks and their semi-trailers, including flatbed, barn-grate, and tipper types, are specified with uniform width and height limits of 2550 mm and 4000 mm, respectively. Manufacturers typically design vehicles to maximize cargo capacity while ensuring safety and stability, often approaching or reaching these size limits. As a result, the average width and height of three-axle, four-axle, and six-axle vehicles are very similar.

**Figure 12:**
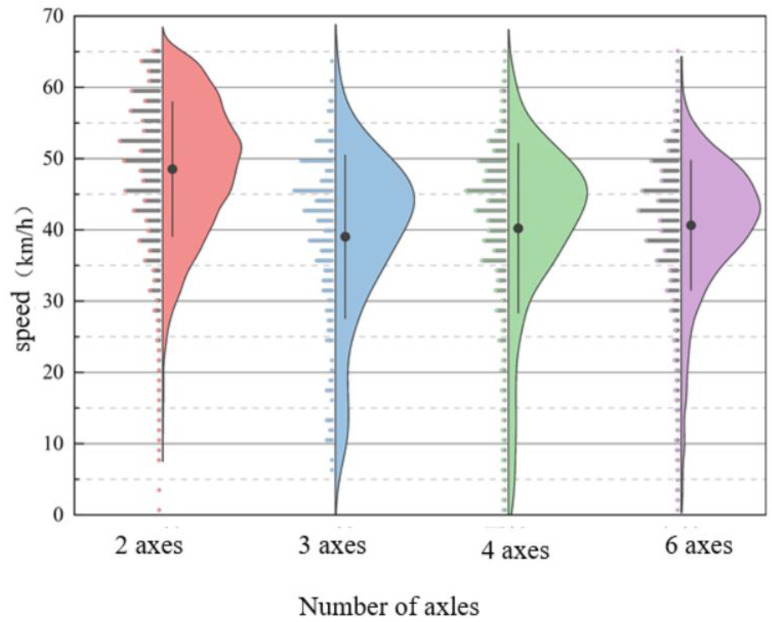
Vehicle speed distribution for different axle types.

Figure 12 shows violin plots of the speed distribution for vehicles with different axle types. Four plots are presented to illustrate the speed distribution for vehicles with varying axle counts. Each plot displays the range of vehicle speeds from the minimum to the maximum value, with a central line indicating the interquartile range of speeds, and a black dot representing the median speed.

As shown in the figure, two-axle vehicles tend to have higher speeds, with their speeds primarily distributed between 40 km/h and 60 km/h, and their maximum speed exceeding that of other vehicle types. Three-axle, four-axle, and six-axle vehicles, which are trucks, generally have speeds ranging from 30 km/h to 50 km/h. The occurrence of zero speeds for some vehicles is due to the presence of a pedestrian crosswalk in front of the dynamic weighing detection point, causing vehicles to stop to yield to pedestrians.

**Figure 13:**
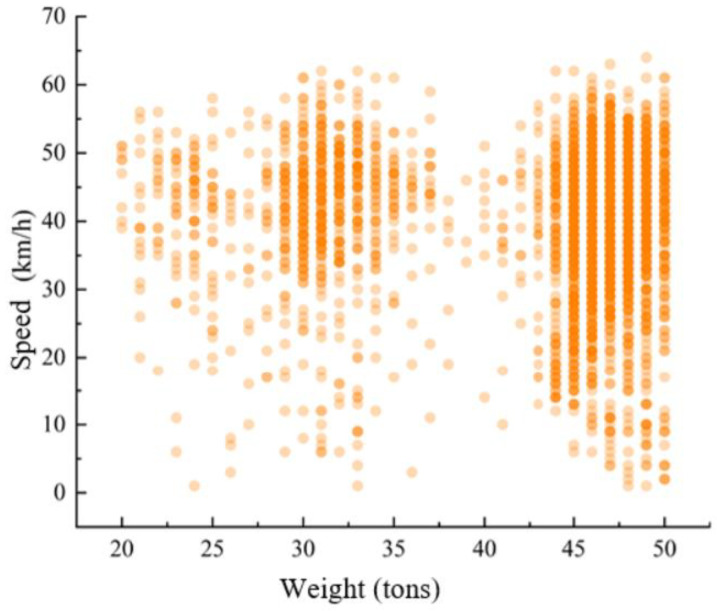
Speed distribution of trucks of different weights.

Figure 13 shows a scatter plot of vehicle speed and total weight for vehicles passing through the dynamic weighing area, with the data collected for various truck types ranging from 20 to 50 tons. The darker the color of the scatter points, the more overlap there is, meaning more vehicles have the same weight and speed. Vehicles weighing between 45 and 50 tons are mostly six-axle trucks. Observing the data, it is clear that the speed of six-axle trucks passing through the dynamic weighing area varies widely.

Since six-axle trucks typically have longer bodies (up to 13 m), their passage through the dynamic weighing zone is more variable. This variability is primarily reflected in changes in the load distribution on each axle during the weighing process and fluctuations in speed. The longer body length of these vehicles causes more noticeable movement in the center of gravity, which significantly affects the accuracy of the dynamic weighing system.

### 4.3. Influence of Different Motion States of Vehicles on Weighing Accuracy and Compensation Experiments

#### 4.3.1. Extraction of Information About Vehicles in Dynamic Weighing Areas

Since the actual weight of vehicles traveling on the road cannot be directly obtained, this study focuses on Jidong cement tanker trucks as the research subject. The company is located in Yaoshu District, Tongchuan City, and some of the cement trucks’ routes pass through the smart super-control site discussed in this paper. After loading cement, the vehicles are weighed using a weighbridge, and the transportation company provided the actual weight and vehicle length for this fleet. The weights of all monitored vehicles were estimated using an average of 17.5 tons, and the length was set at 12.5 m.

**Figure 14:**
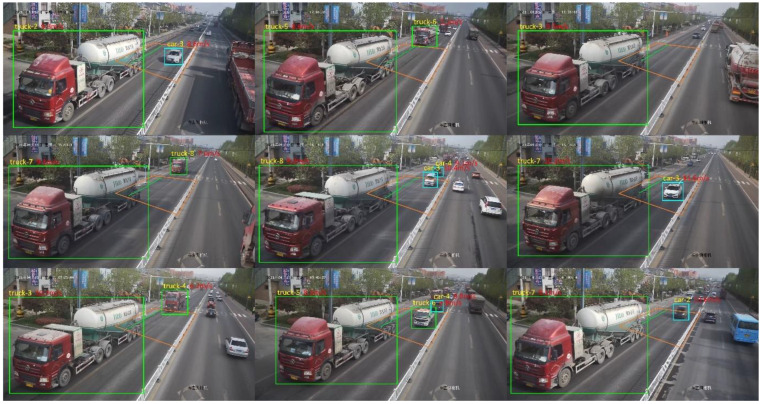
The effect of extracting motion state information of some trucks.

Using the surveillance cameras installed in the dynamic weighing system, motion state information was successfully extracted for 384 vehicles of this type as they passed through the dynamic weighing area. Figure 14 illustrates the extraction results of motion state information for some of the vehicles.

#### 4.3.2. Analysis of the Effect of Different Motion States of Vehicles on Weight Accuracy

**Figure 15:**
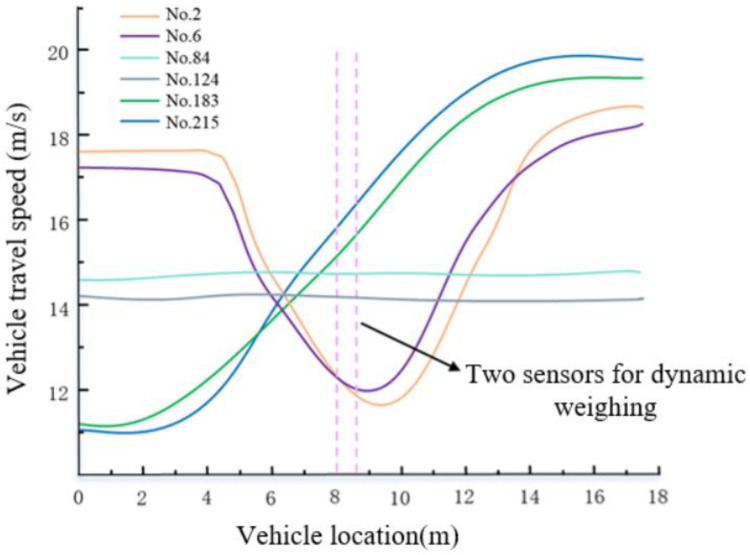
Effect of different motion states.

Figure 15 shows the speed changes of some vehicles as they pass through the dynamic weighing area. The vertical axis represents the vehicle speed extracted from the video, which has been smoothed in this study. The horizontal axis represents the length of the designated motion state extraction area, and the dotted lines in the figure indicate the position of two rows of dynamic load cells within this area. This method allows for a clear visualization of how a vehicle’s motion state changes over time as it moves through the dynamic weighing area, from a distant to a closer position. To facilitate analysis and tracking, the 384 trucks extracted in this study were numbered based on their appearance order in the surveillance video.

In Figure 15, No. 2 represents the graph of the motion state change of truck number 2 as it passes through the dynamic weighing area. Similarly, the other sections of the figure follow the same numbering convention and represent the motion states of trucks with different numbers. A detailed analysis of the video data reveals that the vehicle’s speed exhibits variations while passing through the weighing area. These variations are mainly reflected on the y-axis, showing speed fluctuations between 4 m and 14 m. To further analyze these changes in the motion states of the vehicles, quantitative analyses of velocity and acceleration were conducted on three trucks with different motion states. The results of the analysis are summarized in the table below, which lists the coefficient of variation of velocity and the root mean square of acceleration for each vehicle. It can be observed that both the coefficient of variation of velocity and the root mean square of acceleration reflect the fluctuations in speed. The specific data are shown in Table 6.

After thoroughly classifying and analyzing all the collected data, we found that the trucks passing through the weighing area can be categorized into three distinct movement states: steady driving, accelerating, and decelerating followed by accelerating. These classifications are based on the surveillance video data of 384 trucks and their corresponding weighing records:

(1) When the truck passes through the dynamic weighing area in a steady or accelerating state, the error between the dynamic weighing result and the static weight is kept within 3%. This indicates that the truck’s impact on the dynamic weighing accuracy is relatively small in these two motion states, and the system is capable of accurately measuring the truck’s weight. The main reason for this is that the truck’s center of gravity is generally concentrated near the rear axle, and even during acceleration, the rearward shift of the center of gravity is relatively small, thus limiting its impact on the weighing results. Additionally, the data analysis showed that the vehicle’s trajectory changes during the weighing process had a minimal effect on weighing accuracy. This confirms the crucial role of the trajectory sensor in accuracy calibration. The trajectory sensor can accurately detect the position where the vehicle’s tires make contact with the dynamic load cell, allowing for monitoring of trajectory changes during the weighing process.
(2) The situation differs when a truck passes through the dynamic weighing area while decelerating and then accelerating. In this case, the truck’s center of gravity shifts significantly toward the front axle during the weighing process, leading to an error of 1–4 tons between the dynamic weighing result and the static weight, as shown in Table 4. This error occurs primarily because the center of gravity of the truck is typically located near the rear axle. When the vehicle suddenly decelerates, the center of gravity shifts toward the front axle, causing a substantial negative impact on the weighing accuracy as it passes through the dynamic weighing zone. This finding underscores the importance of considering vehicle motion states in dynamic weighing systems and highlights the need for in-depth studies on how different motion states affect weighing accuracy.

**Figure 16:**
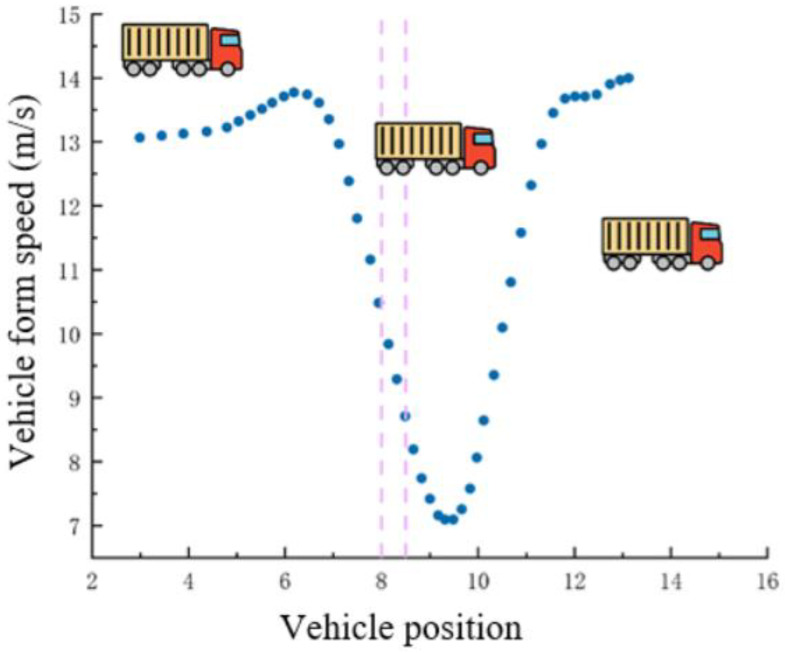
Corresponding effect of position and speed of truck No. 39 on the sensor of dynamic weighing.

In Table 7, truck No. 39 is used as an example. This truck adopted a driving strategy of first decelerating and then accelerating while passing through the dynamic weighing detection area. As shown in Figure 16, the truck gradually slowed down as it approached the dynamic weighing sensor. Due to inertia, the truck’s center of mass shifted toward the front axle [33]. When the front axle of the truck completely passed the sensor, the vehicle’s abrupt deceleration caused the front end to dip, which led to lower load readings from the subsequent three axles, thereby affecting the accurate measurement of the truck’s total weight.

#### 4.3.3. Velocity Characterization

**Figure 17:**
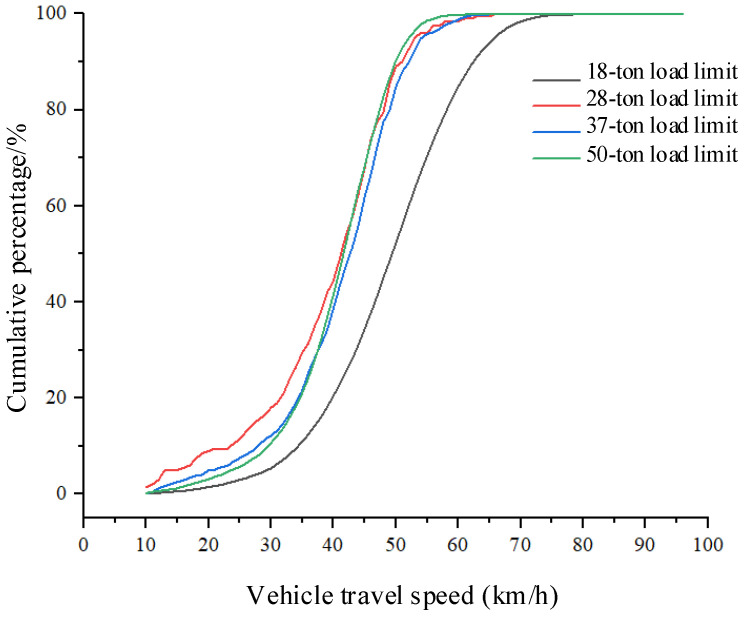
Relationship between vehicle weight limits and speed variations.

**Figure 18:**
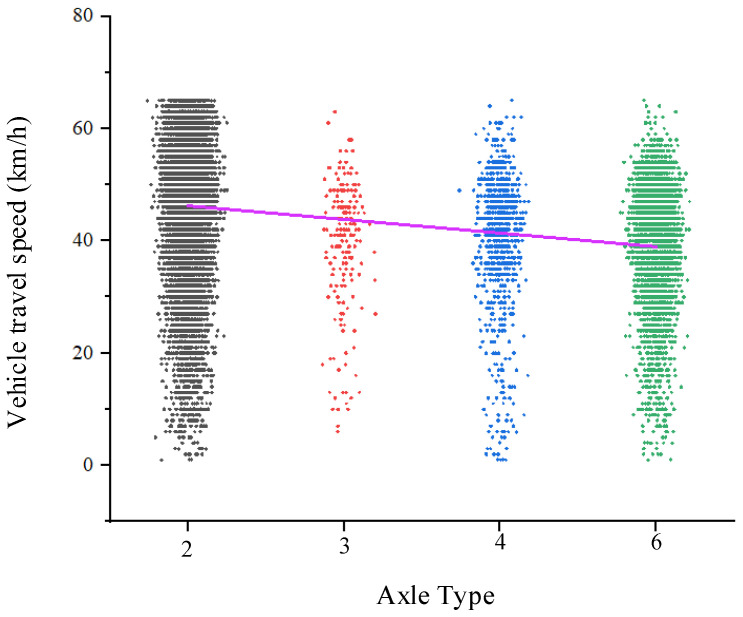
Correlation between different axles and speed distribution.

The dynamic weighing system dataset statistically analyzes the speed distribution of vehicles with different load limits. As shown in Table 8, the distribution includes the average speed, number of vehicles, and average weight for each load limit category. Figure 17 illustrates the speed distribution for vehicles with different load limits. Vehicles with an 18-ton load limit are mostly cars, and their average speed through the dynamic weighing area is generally higher. Vehicles with 28-ton, 37-ton, and 50-ton load limits are mostly three-axle, four-axle, and six-axle trucks, and their speeds through the dynamic weighing area remain consistent, though vehicles with a 28-ton load limit have a slightly higher average speed. Figure 18 presents the speed distribution for vehicles with different axle configurations, showing that the average speed of two-axle cars is higher than that of six-axle trucks. Overall, trucks generally have lower average speeds than cars in the dynamic weighing area. Vehicles with a 44-ton load limit are five-axle trucks, which are not included in the dataset due to their relatively low frequency on the road.

**Figure 19:**
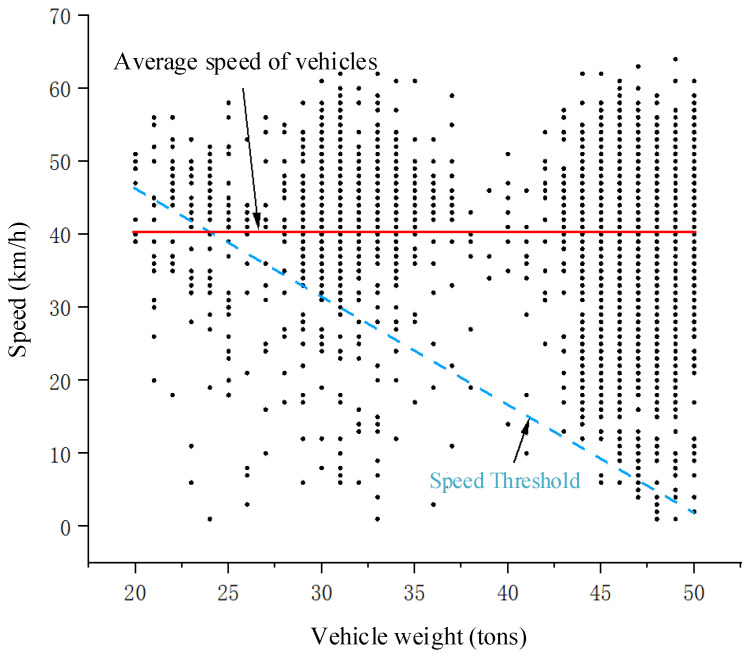
Correlation distribution between vehicle speed and gross vehicle weight (GVW).

Figure 19 shows a scatter plot of the correlation between vehicle speed and gross weight in the dynamic weighing area. The dataset includes only trucks with load limits from 20 to 50 tons, whose average speed in the weighing zone is approximately 40 km/h. The analysis indicates that most vehicles, regardless of weight, travel above the designated speed threshold. Trucks limited to 28 tons maintain speeds of 40–55 km/h; those limited to 37 tons travel at 30–60 km/h; and those limited to 50 tons range from 5–60 km/h. Notably, six-axle trucks exhibit a particularly wide speed range. Their extended length (up to 18.1 m) increases variability in the weighing area since differences in axle loading and shifts in the vehicle’s center of gravity—caused by speed fluctuations during weighing—can significantly affect the dynamic weighing system’s accuracy.

#### 4.3.4. Monte Carlo-Based Data Fusion Analysis

After analyzing the dynamic weighing system dataset, a relationship was identified between the speed of vehicles passing through the dynamic weighing area and their weight. To verify this relationship, this paper creates a comprehensive dataset that merges the dynamic weighing data (including axle weight, total weight, and vehicle speed information), vehicle label and axle type data, and vehicle operating state data (including trajectory and speed change information in the dynamic weighing area). The goal is to analyze the speed change, total weight, and average speed of vehicles passing through the dynamic weighing area.

The data fusion technique known as the Monte Carlo method was employed to create a comprehensive dataset that retains the factors distributed across the sub-datasets. In brief, the Monte Carlo method allows for the presentation of the correlations between key features in the individual datasets [34]. This method uses random sampling to obtain the weights and speeds of vehicles in the dynamic weighing dataset and compares the random sampling results of all weighing data with the vehicle-related information from the vehicle label and axle type dataset, as well as the driving status dataset. If the sampled vehicle speed in the dynamic weighing dataset is below the percentage value of the vehicle speed interval in the driving status dataset, the vehicle is classified into the normal driving group. Conversely, if the sampled vehicle speed exceeds the percentage value in the driving status dataset, the vehicle is classified into the abnormal driving group.

**Figure 20:**
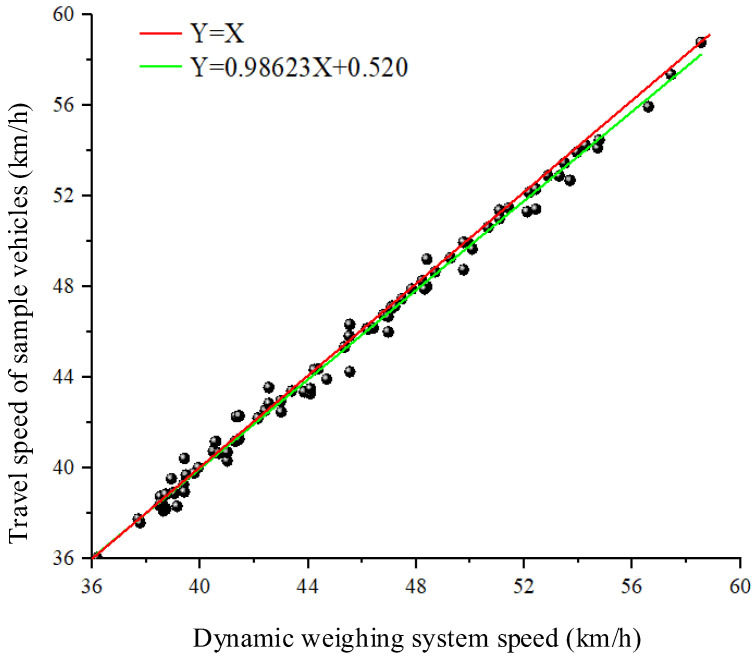
Quartile plot between dynamic weighing system and vehicle traveling state speeds. (Black circles are actual data collection points).

To explore the relationship between the dynamic weighing system dataset and the vehicle traveling state information dataset, quantile statistical plots were created. Figure 20 shows the quantile plots comparing the vehicle speeds in both the dynamic weighing system data and the vehicle traveling state data. Each black dot represents the vehicle speed at each frame as the vehicle passes through the dynamic weighing area, while the horizontal axis represents the average speed of the vehicle through the dynamic weighing area, as measured by the dynamic weighing system. The red line, with a slope of 1, indicates that points along this line represent a speed distribution in the vehicle traveling state data that exactly matches the distribution in the dynamic weighing system. The black dots fluctuate above and below the red line and do not align exactly with it. From this, it can be reasonably inferred that the speed distributions in the dynamic weighing system and the vehicle traveling state data are very similar in shape, with only slight differences in scale and location. This paper uses linear regression to quantitatively describe the relationship, expressed as y = 0.98623x + 0.520. This further demonstrates that the variation in the vehicle’s driving state through the dynamic weighing area, and its effect on weighing accuracy, is related to the vehicle’s driving speed. The final analysis reveals that the correlation between vehicle weight and vehicle speed is stronger than the correlation between vehicle weight and vehicle classification or size.

#### 4.3.5. Compensation Analysis of Weighing Accuracy in Different Motion States of Vehicles

**Figure 21:**
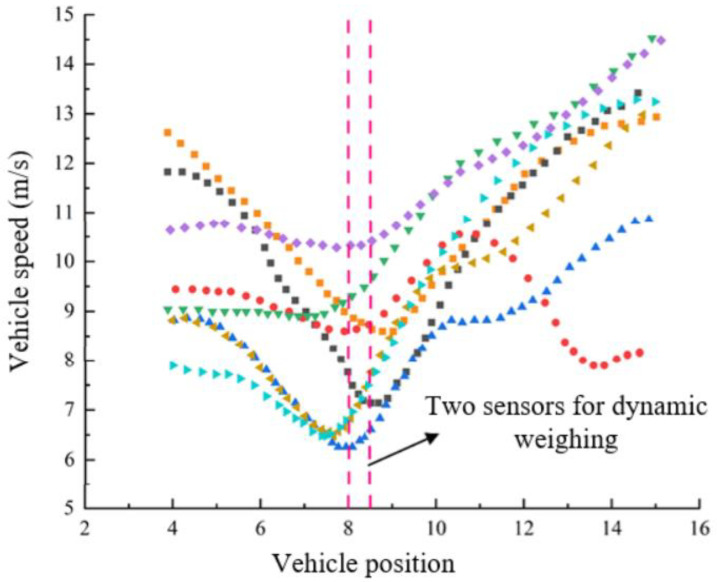
Trajectory and velocity comparison. (Different colors for different cars).

Figure 21 shows a plot of the positions and speeds of eight trucks as they pass through the dynamic weighing detection area, undergoing a process of deceleration followed by acceleration. The two vertical lines in the figure represent the two dynamic load cells installed on the road.

Observation of Figure 21 shows that as the vehicle speed gradually decreases, the points on the trajectory become increasingly compact. Despite this, the vehicle’s path remains largely consistent. Due to the different driving characteristics of various vehicles, their speeds and deceleration behaviors as they pass through the dynamic weighing area vary. A comparative analysis of the collected data reveals that the deceleration behavior of vehicles entering and exiting the dynamic weighing zone significantly impacts the accuracy of the weighing results.

As shown in Figure 21, as the speed decreases, the corresponding trajectory points become denser, but the trajectory remains largely stable. The traveling speeds of different vehicles vary, and the degree of deceleration also differs among vehicles. Data comparison reveals that the impact of deceleration on weighing accuracy varies between the time the vehicle enters and exits the dynamic weighing area.

To accurately compensate for weighing accuracy, a method based on the deceleration ratio is proposed. The data shows that 95% of vehicles have deceleration ratios between 0.09 and 0.5. The formula for calculating the deceleration ratio is as follows:

(29) $$j=\frac{v_{r}}{v}$$

where j denotes the variable speed ratio, $v_{r}$ denotes the degree of variable speed of the vehicle as it passes through the dynamic weighing area, and v denotes the average speed of the vehicle as it passes through the dynamic weighing area.

As shown in Table 9, the driving state data and weighing data of all vehicles passing through the dynamic weighing area under the state of first deceleration and then acceleration are statistically analyzed. The trucks passing through the weighing zone are categorized into three types according to their speeds and the corresponding weighing error ranges, and the number of vehicles with different speed ranges among the detected vehicles is also counted. Accuracy compensation is given for vehicles passing through the weighing zone in different driving states. When such trucks are detected passing through the weighing zone, the dynamic weighing system can compensate for the accuracy according to their driving status, vehicle markings, and axle types, thus realizing personalized weighing calibration and improving the weighing accuracy, which can reduce the weighing error to within 1.8%.

### 4.4. Analysis of the Results of the Validation Experiment

**Figure 22:**
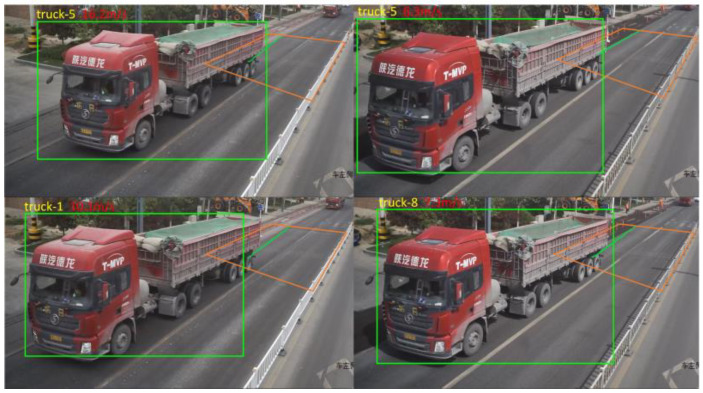
Effect of extracting motion state information of 6-axle trucks.

To evaluate the generalizability of the proposed method, its accuracy in obtaining vehicle motion state information, and its potential impact on dynamic weighing system accuracy correction, a six-axle truck weighing 45.67 tons and approximately 12.5 m long was selected as the subject for experimentation. The truck traversed the dynamic weighing area under various driving conditions for experimental validation. Figure 22 illustrates the extracted motion state information of the six-axle truck while passing through the weighing area.

Consistent with the results of the initial cement tanker experiment, the system provided accurate weighing results when the truck traversed the dynamic weighing area under smooth or accelerated conditions. Even when accelerating, the system maintained high measurement accuracy. This phenomenon can be attributed to two main factors: First, the truck’s center of gravity is typically located near the rear axle, and the spacing between the rear axles is relatively short, usually less than the distance between the load cells. As a result, the center of gravity does not shift significantly during the weighing process, contributing to stable weighing data as the vehicle passes through the area. Second, analysis of the driving data from a large number of vehicles reveals that the acceleration of most vehicles when crossing the dynamic weighing area is limited. This means that the vehicle’s speed change is not too abrupt, which facilitates the accurate capture and processing of weighing information by the system, as shown in Table 10.

The experimental results show that dynamic weighing introduces some error when vehicles pass through the dynamic weighing area at varying speeds. Based on the root mean square error of the vehicle’s acceleration, accuracy compensation can be applied for vehicles with different acceleration and deceleration rates.

The analysis indicates that changes in the vehicle’s motion state do indeed affect the weighing accuracy of the dynamic weighing system. Furthermore, the impact of axle length variations on weighing accuracy differs across vehicles. This suggests that compensating for weighing accuracy based on different vehicles and their motion state changes is crucial for improving the overall system’s accuracy.

## 5. Experimental Verification

### 5.1. Model Training

In this paper, the vehicle’s motion state information extraction detection model is trained using a self-made dataset, and the network training environment is shown in Table 11.

The input image is preprocessed with a size of 300,300 and the batch size is set to 8. For the selection of the optimization algorithm, SGD is used as the optimizer and the training parameters of the model are shown in Table 12.

**Figure 23:**
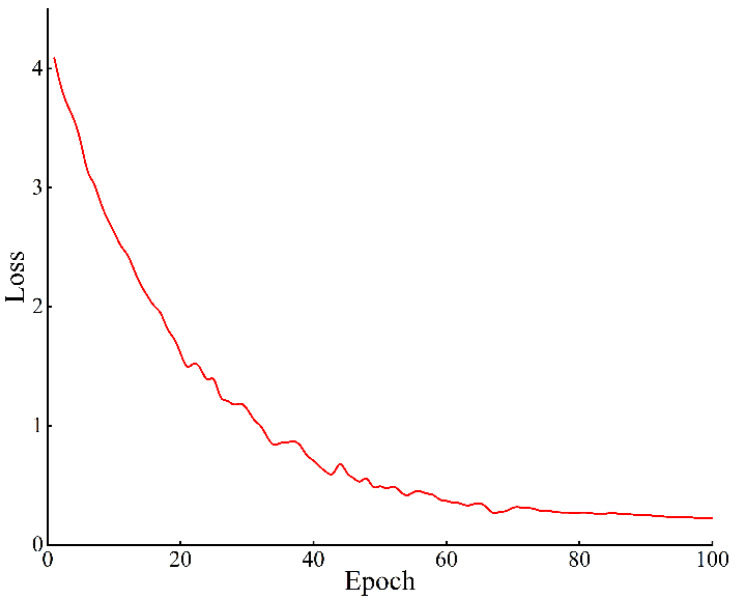
Plot of model training loss function.

During the training of the detection model for vehicle state information extraction, the change in the loss function is a key indicator of whether the model has converged. Figure 23 shows the loss value curve during the training process. The curve reveals that during the first 20 epochs, the model’s training loss decreases rapidly; between epochs 20 and 60, the rate of decrease slows significantly; and from epochs 60 to 100, the loss value stabilizes, indicating that the model has largely completed its training. Table 13 presents the performance of the optimized detection model on the test set. The results demonstrate an improvement in accuracy compared to the pre-optimization network, along with increases in recall rate and frames processed per second. This suggests that the optimized model not only enhances accuracy but also significantly boosts detection speed and real-time performance.

**Figure 24:**
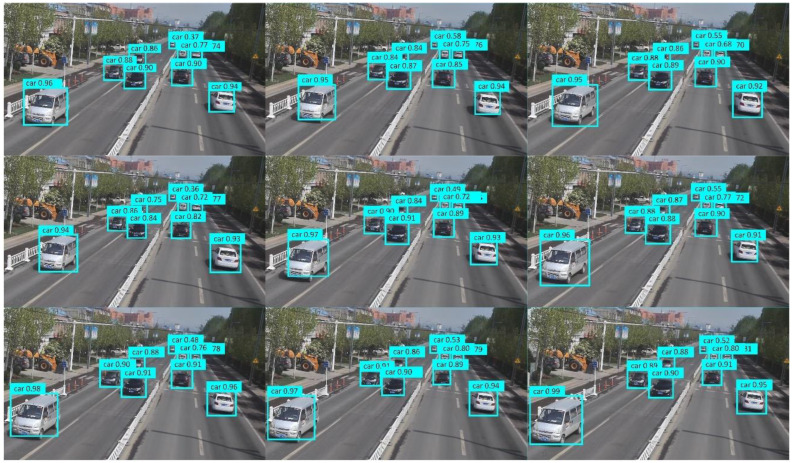
Effect of detecting nine consecutive frames.

During the testing phase, the trained dynamic weighing area vehicle detection model was applied to the test set to assess its performance and accuracy. As shown in Figure 24, the model performed excellently in real-time multi-vehicle detection tasks on actual roadways, accurately identifying all target vehicles without any missed detections.

### 5.2. Analysis of Results

**Figure 25:**
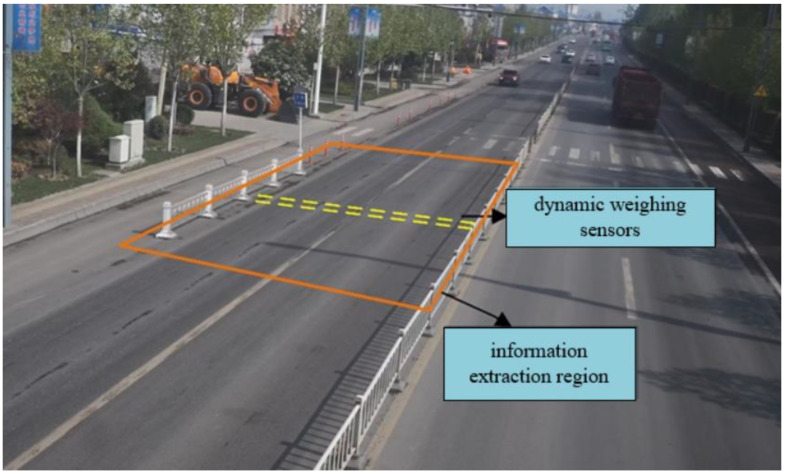
Motion state extraction area setting.

The vehicle motion state information extraction method presented in this chapter aims to accurately capture the trajectory and speed changes of a vehicle as it passes through the dynamic weighing area. Using a detection-based online multi-target tracking method, vehicles on the road are first identified using a target detection algorithm, and then the tracking algorithm is employed to extract their trajectory and speed information. To enhance the accuracy of extracting the trajectory information of vehicles passing through the dynamic weighing area, this paper defines a fixed motion state extraction zone based on field research results. As shown in Figure 25, the dynamic weighing monitoring area is defined as a rectangular zone measuring 8 m by 18 m, with the dynamic weighing sensor positioned at the center of this zone. This method effectively extracts vehicle trajectory and speed change information from monitoring videos, providing crucial data support for the dynamic weighing system.

**Figure 26:**
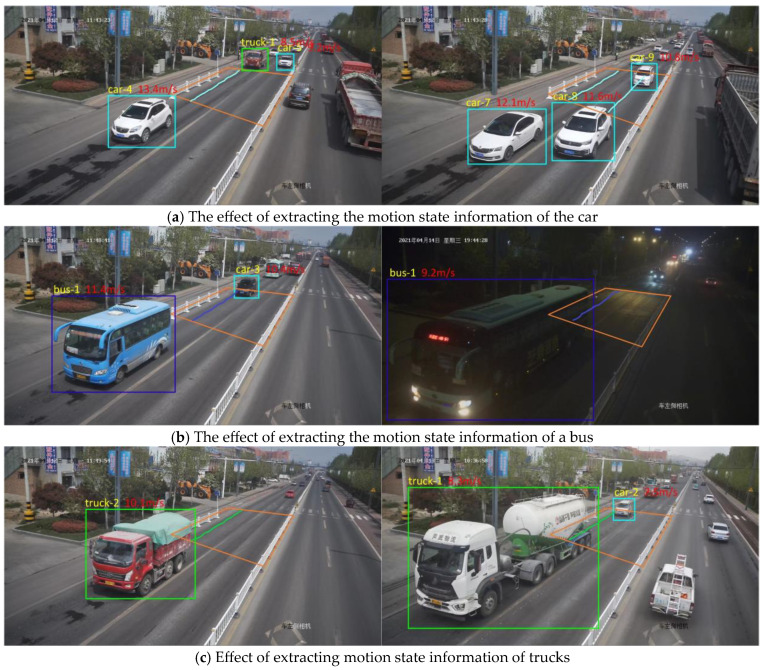
Motion state information extraction results for different types of vehicles.

To verify the effectiveness and reliability of the method proposed in this paper, several different types of vehicles were randomly selected, and their motion state information was extracted within the dynamic weighing area. Figure 26 shows the results of the motion state information extraction for different vehicle types: (a) motion state information for cars; (b) motion state information for buses; and (c) motion state information for trucks. As shown in the figures, even under low light conditions at night, the proposed method accurately captures the motion state information of vehicles passing through the dynamic weighing area. The extraction method in this study focuses on obtaining the motion state information of vehicles within the dynamic weighing area, without detecting or extracting the state of vehicles outside the rectangular zone. This approach not only improves the efficiency of vehicle detection and trajectory tracking but also reduces data processing time, ensuring the real-time nature of the detection process (Figure 26).

## 6. Conclusions

To analyze the impact of vehicle motion state changes on weighing accuracy, it is essential to accurately extract information on vehicle motion state changes within the dynamic weighing area, specifically vehicle position changes and speed fluctuations. By examining the sensitivity of current multi-target tracking methods to vehicle occlusion and the frequent vehicle ID switching during multi-target tracking, this paper proposes a method for extracting vehicle motion state information within the dynamic weighing area. For the detection model, a lightweight SSD model is used, and an optimized loss function is introduced to better suit the dataset characteristics of vehicle motion state information extraction in the dynamic weighing area. This approach effectively enhances target detection stability while maintaining real-time detection performance. The DeepSORT multi-target tracking algorithm is then applied for vehicle trajectory and speed detection, followed by smoothing the vehicle trajectories in the dynamic weighing area using Gaussian Smoothing Interpolation (GSI) technology to obtain smooth and stable vehicle trajectories.

This paper proposes a method for extracting vehicle motion state information in the dynamic weighing area. The extraction of vehicle motion states is essential for analyzing the factors that affect weighing accuracy. To address the issue that the traditional SSD model is highly susceptible to vehicle occlusion, a new loss function is introduced to improve detection accuracy. The DeepSORT algorithm is employed to perform multi-target tracking of vehicles. Initially, Kalman filtering is used for trajectory prediction, and then the Hungarian algorithm, along with cascade matching, is applied to associate detected targets with their corresponding trajectories. To obtain smoother and more stable vehicle trajectories, Gaussian Smoothing Interpolation (GSI) is applied to the output trajectories. Additionally, coordinate transformation is used to convert camera coordinates to real-world coordinates, and an accuracy compensation mechanism is incorporated into the dynamic weighing system. Finally, the proposed vehicle motion state extraction method is validated through experiments. The results demonstrate that the improved vehicle motion state extraction model for the dynamic weighing area not only accurately detects multiple vehicle targets in the video but also shows strong robustness under varying road illumination conditions. The method is capable of effectively detecting vehicle targets in the weighing process video, with minimal impact from vehicle occlusion. Furthermore, it enables faster and more accurate extraction of vehicle motion state information in the dynamic weighing area.

## Figures and Tables

**Table 1:** Distribution of vehicle marking and axle type inspection datasets.

Marker/Axle Type	Quantity/Sheet	Percentage of Data Sets	Marker/Axle Type	Quantity/Sheet	Percentage of Data Sets
dayun	3040	5.41%	zhongqi	2890	5.14%
dongfeng	3510	6.24%	Two axis (2 axles)	14,680	26.12%
futian	3170	5.64%	Three axis (3 axles)	6190	11.01%
shanqi	3890	6.92%	Four axis (4 axles)	4570	8.13%
shangqi	3150	5.60%	Six axis (6 axles)	7920	14.09%
yiqi	3200	5.69%			

**Table 2:** Comparison of different models on homemade dataset.

Methodologies	RMSE/mm	MAE/mm	FLOPs/M	Quantity of Participants/M
YOLOv3	191.43	147.16	1434.65	14.69
MobileNet V1	274.27	243.73	59.96	4.13
GhostNet	252.75	206.84	14.37	5.22
DenseNet	153.48	125.56	2868.44	8.25
Improved DenseNet	98.16	86.62	186.78	5.36

**Table 3:** Comparison of detection results of different detection algorithms.

Parameters	R-CNN	Fair MOT	YOLOv3	The Algorithms in This Paper
Accuracy	73.6%	85.3%	95.2%	96.3%
Recall	78.2%	87.4%	92.4%	96.0%

**Table 4:** Initial eigenvalues of factors after PCA dimensionality reduction.

Factor	Initial Eigenvalue		
	Eigenvalue	Contribution Rate (%)	Cumulative Contribution (%)
speed variation	3.117	31.17	31.17
average speed	2.523	25.23	56.40
Root mean square of acceleration	2.392	23.92	80.32
lengths	1.729	17.29	97.61
widths	0.148	1.48	98.79
heights	0.091	0.91	100

**Table 5:** Distribution of parameters for vehicles with different number of axles.

Vehicle Type	Average Length (m)	Average Width (m)	Average Height (m)	Average Speed (km/h)	Number of Vehicles	Average Weight (tons)
2 axles	5.81	1.97	2.13	47.82	8384	6.31
3 axles	9.21	2.44	3.53	39.04	221	19.65
4 axles	11.45	2.39	3.58	40.22	907	28.93
6 axles	12.67	2.46	3.67	40.64	4291	42.78

**Table 6:** Vehicle movement information sheet.

Vehicle No.	Motion State	Average Speed (m/s)	Velocity Change Factor	Root Mean Square of Acceleration
No.84	uniform velocity	14.68	0.013	0.152
No.2	Slow down first, then speed up.	16.08	0.228	3.24
No.183	speed up	16.28	0.194	3.06

**Table 7:** Comparison of the data of five vehicles passing through the weighing area in the state of deceleration followed by acceleration.

No.	Average Speed (m/s)	CV	RMS	Weight (tons)	Dynamic Weighing Results (tons)	Weighing Error
7	11.84	0.228	3.24	17.5	16.0	8.6%
39	13.25	0.257	3.31	17.5	15.8	9.7%
83	10.46	0.224	3.03	17.5	16.2	7.4%
126	10.63	0.212	2.86	17.5	16.2	7.4%
131	18.73	0.345	3.87	17.5	14.5	17.1%

**Table 8:** Distribution of average speed, number of vehicles and average weight of vehicles under different load limits.

Axle Type	Categorization	Average Speed (km/h)	Number of Vehicles	Average Weight (tons)
2	18-ton load limit	49.10	8539	15.41
3	28-ton load limit	42.98	203	18.85
4	37-ton load limit	40.21	869	26.49
6	50-ton load limit	40.58	5389	43.52

**Table 9:** Vehicle gear ratio corresponding to the weighing accuracy impact and compensation distribution table.

Model No.	Gear Ratio (j)	Weighing Error (tons)	Number of Vehicles	Compensation Accuracy Value (tons)
1	0.09–0.11	1.1	5	1.1
2	0.12–0.13	1.2	9	1.2
3	0.14	1.3	13	1.3
4	0.15–0.16	1.4	28	1.4
5	0.17	1.5	35	1.5
6	0.18–0.19	1.6	29	1.6
7	0.20	1.7	26	1.7
8	0.21	1.8	16	1.8
9	0.22–0.23	1.9	10	1.9
10	0.24	2.0	6	2.0
11	0.25	2.0	13	2.0
12	0.26	2.1	15	2.1
13	0.27	2.2	13	2.2
14	0.28	2.3	11	2.3
15	0.29	2.4	13	2.4
16	0.30	2.5	9	2.5
17	0.31	2.6	11	2.6
18	0.32	2.7	6	2.7
19	0.33	2.8	5	2.8
20	0.34	2.9	2	2.9
21	0.35	3.0	1	3.0
22	0.36–0.38	3.1	9	3.1
23	0.39	3.2	6	3.2
24	0.40–0.43	3.3	7	3.3
25	0.44–0.45	3.4	5	3.4
26	0.46–0.47	3.5	3	3.5
27	0.48–0.49	3.6	2	3.6
28	0.50	4.0	2	4.0

**Table 10:** Experimental vehicle speed fluctuations corresponding to the impact of weighing accuracy and compensation distribution table.

Experiment No.	Axial	Standard Weight (tons)	Detected Weight (tons)	Gear Ratio (j)	Weighing Error (tons)	Accuracy Compensation (tons)
a	6 axles	45.67	43.12	4.28	2.55	2.5
b	6 axles	45.67	42.17	4.64	3.5	3.5
c	6 axles	45.67	44.32	2.97	1.35	1.3
d	6 axles	45.67	45.11	2.16	0.56	0
e	6 axles	45.67	43.86	3.84	1.81	1.8
f	6 axles	45.67	43.65	3.79	2.02	1.8
g	6 axles	45.67	43.20	4.13	2.47	2.2
h	6 axles	45.67	43.90	3.68	1.77	1.7
i	6 axles	45.67	45.21	1.85	0.46	0
j	6 axles	45.67	45.52	1.29	0.15	0

**Table 11:** Network training operating environment.

Project	Versions and Models
systems	Windows 11, 64 bit
CPU	China Lenovo Shenzhen Intel i7-9700 CPU @3.0 Hz
GPU	China Lenovo Shenzhen NVIDIA GeForce RTX 2080 Super, 8 GB
Deep Learning Framework	Pytorch 1.3.0
programming language	Python 3.7.0

**Table 12:** Training parameters.

Parameter Name	Parameterization
Momentum	0.9
weigh_decay	5 × 10^−4^
base_lr	0.0001
lr_policy	poly

**Table 13:** Results of the optimized detection algorithm on the test set.

Comparison Term	SSD Algorithm	Optimized SSD Algorithm
Accuracy	85.2%	93.6%
mAP	84.3%	89.4%
FPS	45.7	66.3

## Data Availability

The original contributions presented in the study are included in the article; further inquiries can be directed to the corresponding author.
